# Life-threatening vancomycin-induced immune hemolytic anemia: A rare case report

**DOI:** 10.1097/MD.0000000000045544

**Published:** 2025-10-31

**Authors:** Jiang-Chen Peng, Yuan Gao

**Affiliations:** a Department of Critical Care, Ren Ji Hospital, School of Medicine, Shanghai Jiao Tong University, Shanghai, China.

**Keywords:** adverse drug reaction, direct antiglobulin test, drug-induced immune hemolytic anemia, life-threatening, vancomycin

## Abstract

**Rationale:**

Drug-induced immune hemolytic anemia is a rare but serious cause of autoimmune hemolytic anemia, most frequently triggered by antimicrobials. Vancomycin is an exceedingly rare culprit, with only a handful of cases reported.

**Patient concerns:**

A 64-year-old woman developed acute-onset fever, tachycardia, dyspnea, and dark-colored urine on the 7th day after revision knee arthroplasty, during which she was receiving intravenous vancomycin prophylaxis.

**Diagnoses:**

Laboratory findings revealed a dramatic drop in hemoglobin, indirect hyperbilirubinemia, elevated lactate dehydrogenase, and reticulocytosis, consistent with hemolysis. The direct antiglobulin test was positive for IgG and C3, confirming immune-mediated hemolysis. A comprehensive workup ruled out infections, autoimmune diseases, and malignancies. The association with vancomycin was categorized as “probable” on the Naranjo scale.

**Interventions:**

Vancomycin was immediately discontinued. The patient received intravenous methylprednisolone and immunoglobulins. Due to rapid progression to life-threatening anemia and hemodynamic instability, therapeutic plasma exchange and washed red blood cell transfusions were initiated.

**Outcomes:**

Following these interventions, the patient’s condition gradually stabilized. Hemoglobin levels improved, hemolytic markers normalized, and she was successfully weaned from mechanical ventilation. She was discharged after 1 month on a prolonged steroid taper, with a full hematologic recovery observed on follow-up.

**Lessons:**

Vancomycin can induce life-threatening immune hemolytic anemia. Clinicians must maintain a high index of suspicion for drug-induced immune hemolytic anemia in patients with abrupt hemolysis after initiating a new drug, as immediate discontinuation of the offending agent is the cornerstone of management.

## 1. Introduction

Autoimmune hemolytic anemia (AIHA) is a form of acquired hemolysis resulting from the immune system mistakenly targeting the body’s own red blood cell antigens, leading to decompensated destruction.^[1]^ In adults, this condition is frequently associated with hematologic malignancies, infections, and autoimmune disorders such as systemic lupus erythematosus, rheumatoid arthritis, scleroderma, and ulcerative colitis.^[2]^ Drug-induced immune hemolytic anemia (DIIHA) is also a well-known but rare cause of anemia. The incidence of DIIHA is approximately 1 per million/yr.^[3]^ The 3 main drug categories associated with DIIHA are: antimicrobials (42%), anti-inflammatory drugs (15%), and antineoplastics (11%).^[4]^ The most common drugs among them are penicillins and cephalosporins.^[5]^ It is essential to recognize this serious adverse drug reaction (ADR), since discontinuing the drug promptly may avoid a deadly consequence.

Here, we reported a case of life-threatening AIHA induced by vancomycin in a 64-year-old woman following revision total knee arthroplasty.

## 2. Case description

A 64-year-old woman with a history of hypertension was admitted to our hospital to undergo right knee arthroplasty. During this hospitalization, her vital signs and physical examination did not reveal any abnormalities. The initial complete blood cell count was normal with white blood cell count of 5.33 × 10^9^/L, a hemoglobin concentration of 12.4 g/dL and a platelet count of 224 × 10^9^/L. Other laboratory indicators related to inflammation, liver, and renal function were all normal. During the surgical procedure, she did not undergo a blood transfusion. Postoperatively, she was administered intravenous (IV) vancomycin at a dosage of 1.0 g every 12 hours as antibiotic therapy.

On postoperative day (POD) 7, the patient suddenly experienced tachycardia, high fever (40 °C) and short of breath. The contrast-enhanced computed tomography showed no significant abnormalities and evidence of bleeding. Then, she was transferred to the intensive care unit (ICU). Upon investigation, physical examination revealed partial oxygen saturation (SpO_2_) of 95%, temperature of 38.5 °C, respiratory rate of 30 breaths/min, heart rate of 110 beats/min, and blood pressure of 144/64 mm Hg. She also featured pale conjunctiva and yellowish skin color. The patient subsequently appeared dark colored urine after inserting urinary catheter and her hemoglobin level was found to drop dramatically from 9.7 g/dL to 4.2 g/dL within 24 hours. Other laboratory findings showed elevated indirect bilirubin, lactate dehydrogenase, and reticulocyte percentage (Table 1). The peripheral blood smear demonstrated spherocytosis. Given high suspicion for immune-mediated hemolytic anemia, vancomycin was discontinued. The patient was started on IV methylprednisolone 1 mg/kg and immune globulin 500 mg/kg.

**Table 1:** Laboratory findings at the suspicion of hemolysis.

Lab test	Value	Reference range
Hemoglobin	42 g/dL	115–150 g/dL
Platelet count	229 × 10^9^/L	125–350 × 10^9^/L
Reticulocyte count	0.028 × 10^12^/L	0.030–0.096 × 10^12^/L
Reticulocyte percentage	13.15%	0.5–1.5%
Total bilirubin	120.8 μmol/L	0–23.0 μmol/L
Indirect bilirubin	63.9 μmol/L	0–17 μmol/L
Lactate dehydrogenase	3870 U/L	120–250 U/L
Haptoglobin	14.4 mg/dL	32–205 mg/dL
Direct Coombs test	Positive (IgG, C3)	Negative

C3 = complement 3.

**Figure 1.**
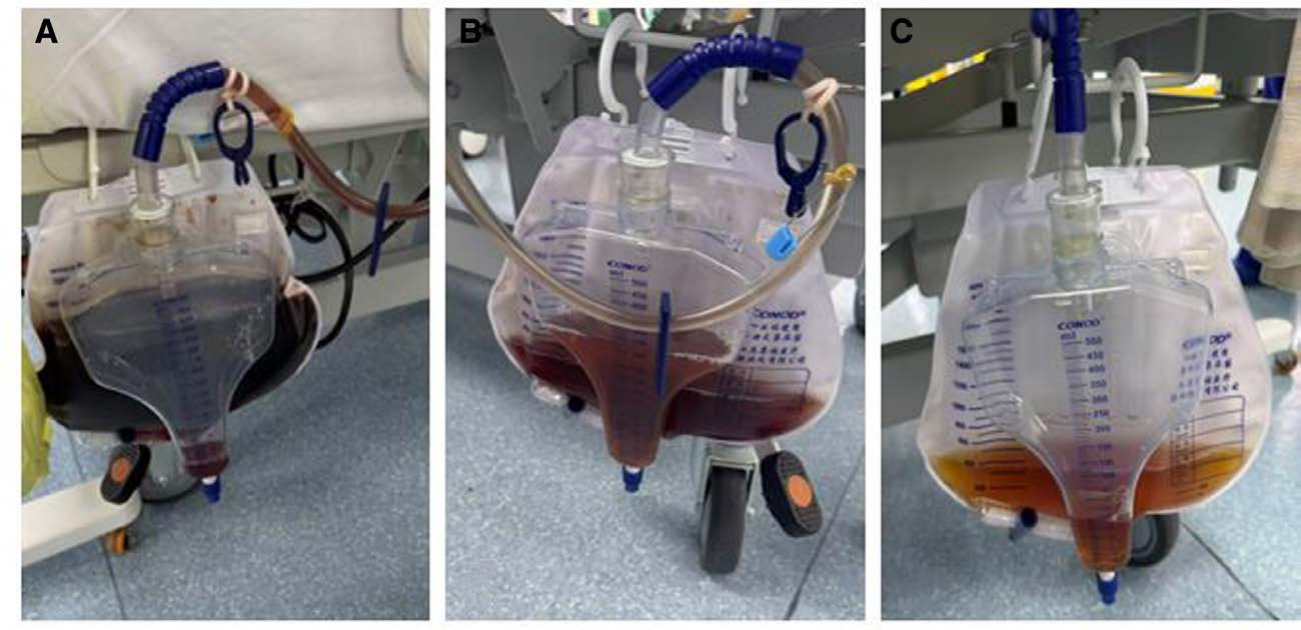
The patient’s dark-colored urine gradually lightened to yellow following therapeutic plasma exchange (TPE). (A) Urine color before TPE; (B) urine color 2 days after TPE; (C) urine color 4 days after TPE.

**Figure 2.**
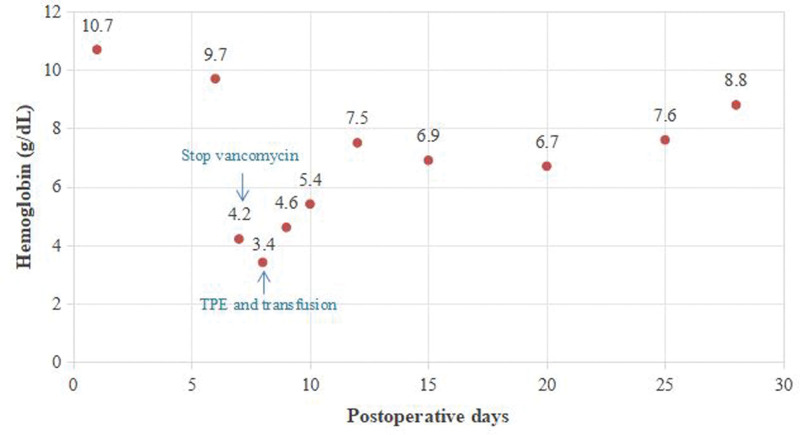
The dynamic change of hemoglobin after operation. TPE = therapy plasma exchange.

On the morning of POD 8, the patient developed critical anemia with hemoglobin plummeting to 3.4 g/dL. She exhibited severe hypoxemia (SpO_2_ < 85%), hemodynamic instability (blood pressure 80/40 mm Hg), and metabolic derangement (lactate 10.0 mmol/L), necessitating emergent endotracheal intubation for respiratory support and vasopressor therapy to maintain perfusion pressure. Subsequently, the direct antiglobulin test (DAT) revealed that the patient’s red cells were coated with both IgG and complement 3 (C3). Therapeutic plasma exchange with 2000 mL frozen plasma were immediately performed for the severe anemia and persistent hemolysis. Besides, 4 units of washed red blood cells (RBCs) were transfused. Over the next 48 hours, the patient demonstrated marked clinical improvement with reduction of lactate levels, stabilization of hemoglobin and resolution of hemoglobinuria (Fig. 1). On POD 12, the patient was successfully extubated. After 14 days of steroid use, methylprednisolone was gradually tapered at a rate of 10 mg per week. Throughout 1-month hospitalization, the hemoglobin level improved to 88 g/dL (Fig. 2) and hemolytic laboratory markers showed significant improvement. She was ultimately discharged on a prolonged oral methylprednisolone taper regimen.

Initially, the etiology of AIHA remained unclear. The next-generation sequencing of both synovial fluid and blood yielded negative results. Viral serology testing for human immunodeficiency virus, hepatitis B and C viruses, Epstein-Barr virus, and cytomegalovirus returned negative findings. Additionally, autoimmune evaluations including antinuclear antibodies, anticardiolipin antibody, anti-neutrophil cytoplasmic antibodies, C3, complement 4, and rheumatoid factor were all within normal limits. Furthermore, lymphoproliferative disorders were ruled out based on normal immunoglobulin levels (IgG, IgM, and IgA) and unremarkable serum protein electrophoresis results. Besides, serum tumor biomarker testing also yielded negative results (Table 2). As demonstrated in Table 3, a strong association was observed between the DAT positivity and vancomycin administration. Following discontinuation of the drug, the DAT reactivity for both anti-IgG and anti-C3d gradually weakened, turning negative by day 8 after cessation. The Naranjo Adverse Drug Reaction Probability Scale was applied to assess the likelihood of an adverse drug reaction, with results categorized as definite, probable, possible, or doubtful.^[6]^ Based on the calculated score of 6, the association between vancomycin and hemolytic anemia was categorized as “probable.” On 1-month follow-up, the hemoglobin level was noted to be 11.1 g/dL, representing an increase from discharge. Subsequently, the level plateaued at 12.0 g/dL by the 2-month visit.

**Table 2:** Key diagnostic laboratory findings in the workup of hemolytic anemia.

Variable	Result	Reference range
Immunology		
Immunoglobulin A (IgA, g/L)	1.53	1.0–4.2
Immunoglobulin G (IgG, g/L)	17.2	8.6–17.4
Immunoglobulin M (IgM, g/L)	0.72	0.5–2.8
Antinuclear antibodies (ANA)	Negative	Negative
Anti-dsDNA (IU/mL)	30.39	<77
Complement 3 (C3, g/L)	0.978	0.7–1.4
Complement 4 (C4, g/L)	0.194	0.1–0.4
Anticardiolipin antibody IgA (U/mL)	1.60	<12
Anticardiolipin antibody IgG (U/mL)	1.90	<12
Anticardiolipin antibody IgM (U/mL)	1.27	<12
Rheumatoid factor IgA (U/mL)	2.0	<12
Rheumatoid factor IgG (U/mL)	3.7	<12
Rheumatoid factor IgM (U/mL)	2.5	<12
Anticyclic citrullinated peptides (anti-CCP)	0.33	<0.95
Antineutrophil cytoplasmic antibody (ANCA)	Negative	Negative
Pathogen tests		
Epstein-Barr virus (EBV) DNA (copies/mL)	<400	<400
Cytomegalovirus (CMV) DNA (copies/mL)	<400	<400
Hepatitis B surface antigen (HBsAg)	Negative	Negative
Hepatitis C virus (HCV) antibody	Negative	Negative
Human immunodeficiency virus (HIV) antibody	Negative	Negative
Serum tumor markers		
Alpha-fetoprotein (AFP, ng/mL)	6.06	0–7
Carcinoembryonic antigen (CEA, ng/mL)	2.88	0–4.7
CA 19-9 (U/mL)	26.5	0–27
CA 50 (IU/mL)	24.3	0–25
CA 242 (U/mL)	3.15	0–10
CA 125 (U/mL)	32.5	0–35
CA 15-3 (U/mL)	20.4	0–25
CA 72-4 (U/mL)	1.18	0–6.9
Squamous cell carcinoma antigen (SCC-Ag, ng/mL)	2.45	0.5–2.7
Nerve-specific enolase (NSE, ng/mL)	15.8	0–16.3

**Table 3:** Results of the direct antiglobulin test after discontinuation of vancomycin.

After stopping the administration of vancomycin (d)	Antihuman IgG antibody	Antihuman C3 antibody
0	++	++
1	++	++
2	++	+
3	Not tested	Not tested
4	Not tested	Not tested
5	++	−
6	+	−
7	+	−
8	−	−

C3 = complement 3.

## 3. Discussion

Although DIIHA is rare, with an estimated incidence of 1 in a million cases, its prevalence is likely underestimated. This is because only the most severe cases typically undergo the necessary investigations to confirm a drug-induced etiology.^[3]^ According to the International Consensus on AIHA Diagnosis and Treatment, DIIHA is recognized as a secondary cause of AIHA and should be systematically ruled out in suspected cases.^[7]^ To date, more than 130 drugs have been implicated in triggering immune-mediated hemolysis.^[8]^ The hallmark of DIIHA is a sudden and significant drop in hemoglobin levels following drug initiation. The clinical manifestations were hemolytic anemia with a clear time correlation with drug administration. In severe cases, rapid hemolysis can lead to life-threatening complications, including hypovolemic shock and cardiopulmonary arrest, as observed in our patient. Given the potential for rapid clinical deterioration, prompt diagnosis is critical to ensure timely discontinuation of the causative drug.

The mechanism of DIIHA is due to the immune damage to RBCs caused by drug-induced antibodies, including drug-dependent antibodies and drug-independent antibodies.^[9]^ In drug-dependent antibodies, pharmaceutical compounds function as haptens that conjugate with RBCs’ membranes or serum proteins to form holoantigens. The resulting antibodies (typically of the IgG class) specifically target drug-coated RBCs, leading to their immune-mediated destruction through binding to the drug antigens present on the erythrocyte surface.^[10]^ In severe cases, the immune complex formed by the drug, erythrocyte membrane proteins, and antidrug antibodies binds nonspecifically to the erythrocyte membrane and activates the complement system. This typically results in a precipitous decline in hemoglobin levels accompanied by the abrupt onset of clinical symptoms.^[11]^ As demonstrated in this case, the DAT for both IgG and C3 led to a critically low hemoglobin level of 3.4 g/dL, resulting in acute life-threatening complications. Drug-independent antibodies are antibodies that, upon exposure to certain medications, can induce modifications in the antigenic epitopes of RBCs. These altered epitopes stimulate the immune system to produce antibodies targeting the patient’s own RBCs, ultimately leading to immune-mediated hemolytic anemia.^[9]^ Patients may exhibit mild to moderate anemia, while severe anemia is less common.

Vancomycin, a polycationic glycopeptide antibiotic, exerts its bactericidal effect by inhibiting cell wall synthesis in Gram-positive organisms. This agent is associated with various ADRs such as interstitial nephritis, toxic epidermal necrolysis and necrotizing cutaneous vasculitis. Additionally, vancomycin has also been documented to induce immune thrombocytopenia through the formation of vancomycin-dependent antiplatelet antibodies.^[12]^ The well-known and severe ADR to vancomycin is drug reaction with eosinophilia and systemic symptoms, which typically presents with fever, eosinophilia, skin rash, lymphadenopathy, and multiorgan failure. Genetic predisposition, polypharmacy and underlying autoimmune diseases are significant factors that contribute to the pathogenesis of vancomycin-induced drug reaction with eosinophilia and systemic symptoms.^[13]^ Regarding vancomycin-associated DIIHA, it is exceedingly rare with only a handful cases reported in the literature.^[14–16]^ Gniadek et al noted their case was only the second reported instance of vancomycin-induced immune hemolytic anemia. The patient was a 61-year-old woman with a knee prosthesis infection who received vancomycin via IV administration. She developed hemolytic anemia 6 days after IV vancomycin initiation. Cessation of vancomycin led to resolution of hemolysis and specialized serologic testing confirmed the presence of a vancomycin-dependent antibody.^[14]^ Two additional cases were diagnosed clinically. Sudhakaran et al described a 72-year-old male with a prosthetic hip infection who was treated with IV vancomycin and a vancomycin-impregnated spacer. Hemolysis developed approximately 2 weeks after antibiotic initiation. Notably, hemolysis persisted despite discontinuation of systemic vancomycin, likely due to continued local elution of the drug from the spacer. Complete resolution occurred only after surgical removal of the spacer.^[15]^ Siddiqui et al presented a 75-year-old male with recurrent knee infections. The onset of hemolytic anemia occurred within a week after IV vancomycin treatment and hemoglobin stabilized 3 days after vancomycin discontinuation.^[16]^ In the present case, the patient developed acute-onset hematuria accompanied by a dramatic hemoglobin decline following 1 week of IV vancomycin therapy after right knee arthroplasty. The ICU physician promptly identified the potential for DIIHA and immediately discontinued vancomycin. The DAT turned negative after stopping vancomycin for 8 days. Given the close temporal association between vancomycin initiation, the onset of hemolysis, and the subsequent resolution of hemolysis upon discontinuation, vancomycin-induced immune hemolytic anemia was also strongly suspected.

The cornerstone of DIIHA management is immediate drug withdrawal, which critically determines patient prognosis. Patients with DIIHA required to be transferred to the ICU for optimal supportive care. In severe cases like ours, therapeutic plasma exchange should be considered as adjunctive therapy. Complete blood counts should be closely monitored, with expected hemoglobin recovery typically occurring within 1 to 2 weeks after drug discontinuation.^[2]^ The primary limitation of this study was our inability to perform vancomycin antibody testing due to insufficient laboratory resources at our institution, which precluded the assessment of causality. Furthermore, as this report is based on a single patient, the findings lack generalizability and cannot be used to establish broad clinical guidelines.

## 4. Conclusion

Vancomycin-induced hemolytic anemia represents a rare form of DIIHA. When patients present with typical manifestations of hemolytic anemia after starting a new drug, clinicians should maintain a high index of suspicion for this potential complication and pursue comprehensive laboratory investigation. Importantly, prompt discontinuation of the suspected causative agent, particularly antibiotics, is essential for the management.

## Author contributions

**Writing – original draft:** Jiang-Chen Peng.

**Writing – review & editing:** Yuan Gao.
